# Transgenic Tg(Kcnj10-ZsGreen) Fluorescent Reporter Mice Allow Visualization of Intermediate Cells in the Stria Vascularis

**DOI:** 10.21203/rs.3.rs-3393161/v1

**Published:** 2023-10-04

**Authors:** Dillon Strepay, Rafal T. Olszewski, Sydney Nixon, Soumya Korrapati, Samuel Adadey, Andrew J. Griffith, Yijun Su, Jiamin Liu, Harshad Vishwasrao, Shoujun Gu, Thomas Saunders, Isabelle Roux, Michael Hoa

**Affiliations:** Auditory Development and Restoration Program, National Institute on Deafness and Other Communication Disorders, National Institutes of Health; Auditory Development and Restoration Program, National Institute on Deafness and Other Communication Disorders, National Institutes of Health; Auditory Development and Restoration Program, National Institute on Deafness and Other Communication Disorders, National Institutes of Health; Auditory Development and Restoration Program, National Institute on Deafness and Other Communication Disorders, National Institutes of Health; Auditory Development and Restoration Program, National Institute on Deafness and Other Communication Disorders, National Institutes of Health; Otolaryngology Branch, National Institute on Deafness and Other Communication Disorders, National Institutes of Health; Advanced Imaging and Microscopy Resource, National Institutes of Health; Advanced Imaging and Microscopy Resource, National Institutes of Health; Advanced Imaging and Microscopy Resource, National Institutes of Health; Auditory Development and Restoration Program, National Institute on Deafness and Other Communication Disorders, National Institutes of Health; Transgenic Animal Model Core, Biomedical Research Core Facility, University of Michigan; Otolaryngology Branch, National Institute on Deafness and Other Communication Disorders, National Institutes of Health; Auditory Development and Restoration Program, National Institute on Deafness and Other Communication Disorders, National Institutes of Health

## Abstract

The stria vascularis (SV) is a stratified epithelium in the lateral wall of the mammalian cochlea, responsible for both endolymphatic ion homeostasis and generation of the endocochlear potential (EP) critical for normal hearing. The SV has three layers consisting predominantly of basal, intermediate, and marginal cells. Intermediate and marginal cells form an intricate interdigitated network of cell projections making discrimination of the cells challenging. To enable intermediate cell visualization, we engineered by BAC transgenesis, reporter mouse lines expressing ZsGreen fluorescent protein under the control of *Kcnj10* promoter and regulatory sequences. *Kcnj10* encodes KCNJ10 protein (also known as Kir4.1 or Kir1.2), an ATP-sensitive inwardly-rectifying potassium channel critical to EP generation, highly expressed in SV intermediate cells. In these transgenic mice, ZsGreen fluorescence mimics *Kcnj10* endogenous expression in the cochlea and was detected in the intermediate cells of the SV, in the inner phalangeal cells, Hensen’s, Deiters’ and pillar cells, in a subset of spiral ganglion neurons, and in glial cells. We show that expression of the transgene in hemizygous mice does not alter auditory function, nor EP These transgenic Tg(*Kcnj10*-ZsGreen) mice allow live and fixed tissue visualization of ZsGreen-expressing intermediate cells and will facilitate future studies of stria vascularis cell function.

## Introduction

**Figure 1:**
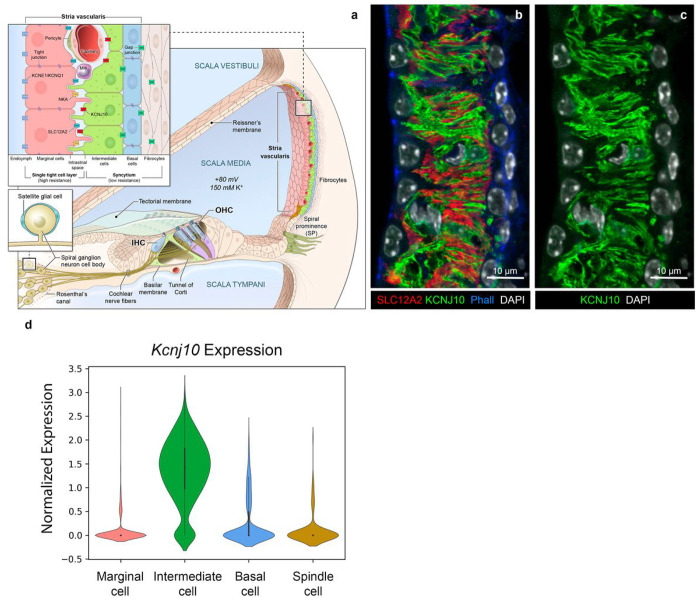
Stria vascularis cellular organization and interdigitation of intermediate and marginal cells **a,** Illustration of the stria vascularis (SV) in relation to the other structures of the cochlear duct. The SV maintains high potassium concentration within the scala media and generates the 80 to 100 mV endocochlear potential (EP), which are both essential for sensory hair cell function and hearing. The SV is comprised of three main layers of cells, the marginal cells (pink, MC), the intermediate cells (green, IC), and the basal cells (blue, BC), which work together to regulate the ionic composition of the endolymph. The intermediate cells have bidirectionally cellular projections that interdigitate with the other two main cell types. Other cells also present in the SV include endothelial cells, pericytes, and macrophages, and in the periphery, spindle cells (gold) close to the Reissner’s membrane and the spiral prominence. Root cells (dark olive green) are present in the spiral prominence. Pillar cells (light green) and Deiters’ cells (lavender) are adjacent and under the outer hair cells (OHCs), respectively. Type I spiral ganglion neurons innervate the inner hair cells (IHC) and type II spiral ganglion neurons innervate the OHCs. Satellite glial cells (turquoise) surround the cell bodies of the spiral ganglion neurons. **b,** Cross-section of the SV in a postnatal day 30 mouse, immunolabeled with anti-KCNJ10 (green) and anti-SLC12A2 (red) antibodies labeling the plasma membrane of the intermediate cells, and marginal cells, respectively. Phalloidin (Phall, blue) labels the F-actin rich tight junctions in the basal and marginal cell layers. DAPI (white) labels all cell nuclei. The extreme interdigitation between the cellular processes of the intermediate and marginal cells can be appreciated. **c,** Same image as Fig. 1b showing only KCNJ10 and DAPI labeling. **d,** Violin plot of *Kcnj10* mRNA relative expression (normalized counts) in the different cell types of the stria vascularis studied at P30 using single nucleus RNA-Seq (data set reported by Gu et al., 2020^3^). *Kcnj10* mRNA is highly expressed in the intermediate cells with minimal expression in marginal, basal and spindle cells.

The stria vascularis (SV) is a stratified epithelial tissue in the lateral wall of the cochlear duct, responsible for ion transport, in particular potassium, into the endolymph-containing scala media. The processes that lead to and maintain an elevated potassium concentration in the endolymph are necessary for the generation and maintenance of an endocochlear potential (EP) of 80 to 100 mV, which is critical for normal hearing^1,2^. The SV contains three layers of main cell types: the basal cells, which are adjacent to the fibrocytes of the spiral ligament, the marginal cells, which directly border the scala media and are in direct contact with the endolymph, and the intermediate cells between these two cell types (Fig. 1a). While the basal cells are easily distinguishable from the other cell types, the intermediate and marginal cells form an interdigitated network of cell projections within the SV^3–5^ (Fig. 1b, 1c). This intricate interdigitation presents a challenge to delineate intermediate cells from the surrounding cells of other types, hindering efforts to identify changes in their number, morphology, ion exchanger and channel composition for examples. Being able to readily identify these cells would facilitate their characterization and the refinement of our understanding of the roles they play in generating the EP and other mechanisms they may be involved in, in both physiological and pathological conditions. Highlighting the importance of studying the physiology of the SV, decreased hearing function has been linked to pathological changes of the SV associated with Alport syndrome^6^, Pendred syndrome^7,8^, Norrie disease^9^, neuraminidase 1-deficiency^10^ and some forms of nonsyndromic deafness^11,12^.

KCNJ10 also known as Kir4.1 and Kir1.2, is encoded by the gene *Kcnj10* in mice. It is an ATP-sensitive inwardly rectifying potassium channel critical to EP generation, highly expressed in SV intermediate cells where it is found concentrated at the plasma membrane of their digitations in contact with the marginal cells (Fig. 1c, d)^1,13,14^. Notably, single-nucleus RNA-sequencing data previously generated by our group also demonstrated the specificity of the expression of *Kcnj10* mRNA in the intermediate cells as compared to the other cell types of the SV (Fig. 1d). As previously reviewed by Chen and Zhao, *Kcnj10* is also expressed in the Deiters’ cells surrounding the outer hair cells in the organ of Corti, and in the satellite glial cells of the cochlear spiral ganglion^13^.

To enable live visualization and fixed tissue identification of the intermediate cells of the SV and their digitations, we generated bacterial artificial chromosome (BAC) transgenic mouse reporter lines expressing cytoplasmic ZsGreen fluorescent protein under the control of the *Kcnj10* promoter region and regulatory sequences. We show that in these reporter mice, ZsGreen fluorescence indeed parallels *Kcnj10* endogenous expression and does not alter auditory function nor EP These reporter mice are a new tool to study the SV at the cellular level and further define its functions.

## Results

Generation of transgenic B6.Cg-Tg(*Kcnj10*-ZsGreen)^skMH^ [Tg(*Kcnj10*-ZsGreen)] fluorescent reporter mice.

**Figure 2:**
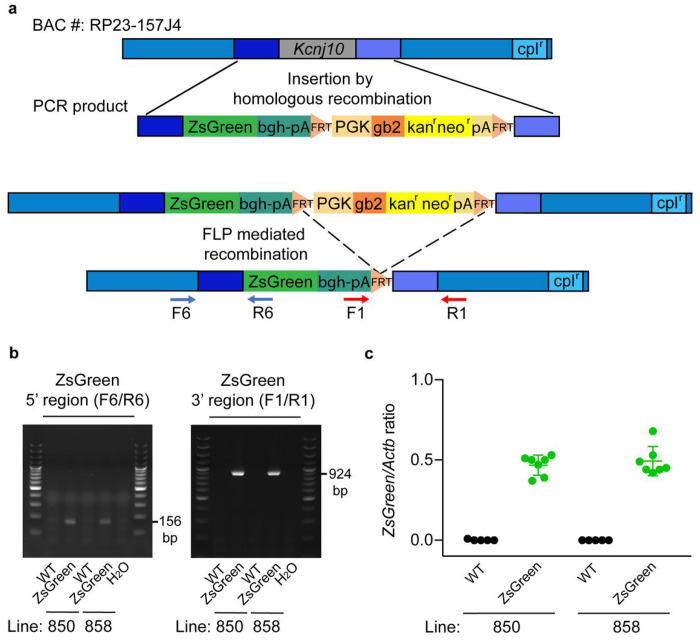
Generation of B6.Cg-Tg(*Kcnj10*-ZsGreen)^skMH^ (*Kcnj10*-ZsGreen) fluorescent transgenic mice. **a,** A schematic of the BAC clone RP23-157J4 expected to contain mouse genomic DNA (gDNA) including promoter, coding sequence and regulatory elements responsible for the endogenous expression of *Kcnj10*, is shown on the top of the panel. The BAC backbone contains a gene of resistance to chloramphenicol (cpl^r^). The BAC and a synthetic donor DNA containing the coding sequence of ZsGreen followed by bgh-polyA(pA) sequences, and a gene conferring kanamycin/neomycin resistance (kan^r^/neo^r^) surrounded by FRT sites, were combined in recombineering competent bacteria. The insertion of this synthetic donor in place of the coding sequence of *Kcnj10* by homologous recombination was made possible by the insertion in 5’ and 3’ of this cassette of 80 bp fragments of DNA homologous to the target regions in the BAC (darker blue regions). Introduction of the cassette into the BAC resulted in kan^r^ of the bacteria. DNA from kan^r^ BACs were further analyzed to identify correctly modified BACs. The kan^r^ cassette has both prokaryotic and eukaryotic (mouse *Pgk 1*) promoters. Black lines indicate regions of recombination between mouse gDNA in the BAC DNA and homologous sequences introduced by PCR in the synthetic DNA donor plasmid. The kan^r^/neo^r^ cassette was removed by expression of flipase (FLP) recombinase. DNA from kanamycin sensitive BACs were analyzed to identify correctly modified BACs. A final recombination step replaced the BAC-backbone internal loxP site with ampicillin resistance (step not shown). **b,** Mice carrying *Kcnj10*-ZsGreen transgene were identified by PCR. F6/R6 primer pair targets the DNA region between the promotor of *Kcnj10* and the 5’ sequence of ZsGreen. F1/R1 primer pair targets the 3’ sequence of ZsGreen and its polyA region. Amplicons of 156 bp with primers F6/R6 and 924 bp with primers F1/R1 indicate the presence of the transgene. These amplicons were detected in *Kcnj10*-ZsGreen mice gDNA but not in the gDNA of their wildtype littermates. The sequence of these primers is reported in Table S10. H_2_O: negative control without gDNA template. DNA size marker (ThermoScientific GeneRuler 100 bp Plus DNA ladder SM0323, Waltham, MA) was used. **c,** To evaluate *Kcnj10*-ZsGreen transgene copy number, ddPCR was performed on the gDNA of ZsGreen hemizygote mice from the founder lines 850 and 858 and their wildtype (WT) littermates. The gene *Actb* encoding b actin, located on chromosome 5 in mice, was used as a reference. ddPCR revealed the presence of ZsGreen targeted amplification in half as many droplets as *Actb* positive droplets, supporting the fact that hemizygous 850 and 858 ZsGreen mice each only carry one copy of the ZsGreen transgene.

To generate *Kcnj10*-ZsGreen transgenic mice, a modified BAC (RP23-157J4) expected to contain *Kcnj10* promoter and regulatory sequences was obtained. A ZsGreen expression cassette was used to replace *Kcnj10* mouse coding sequence (genomic region corresponding to NM_001039484.1: exon 2 nucleotide 243 to 1393) by BAC recombineering approach (Fig. 2a). Before pronucleus injection, the modified BAC was sequenced across ZsGreen to verify that the intended insertion of ZsGreen occurred. The absence of gross recombination inside the BAC was checked by restriction enzyme digestion followed by pulsed field gel.

Four founder mice were obtained. After one breeding with C57BL/6J mice, their progeny was studied for the presence of the transgene, leading to the isolation of four transgenic mouse lines. Two of these, lines 850 and 858 (Fig. 2b), were studied in detail for their ZsGreen expression in the cochlea. These lines were backcrossed for over 10 generations on a C57BL/6J background, to obtain congenic mice.

As BAC transgenes are sometimes inserted in multiple copies, often in tandem, we aimed to identify the number of copies of the ZsGreen transgene in these two mouse lines. To do so, we used digital droplet PCR (ddPCR) to study the gDNA of mice from these two lines and their wildtype littermates. We compared the number of droplets positive for ZsGreen and for the autosomal gene *Actb* used as a reference. As shown in Fig. 2c, hemizygous *Kcnj10*-ZsGreen mice from lines 850 and 858 both have one copy of the ZsGreen transgene.

### Overview of *Kcnj10*-ZsGreen expression in the cochlea.

**Figure 3:**
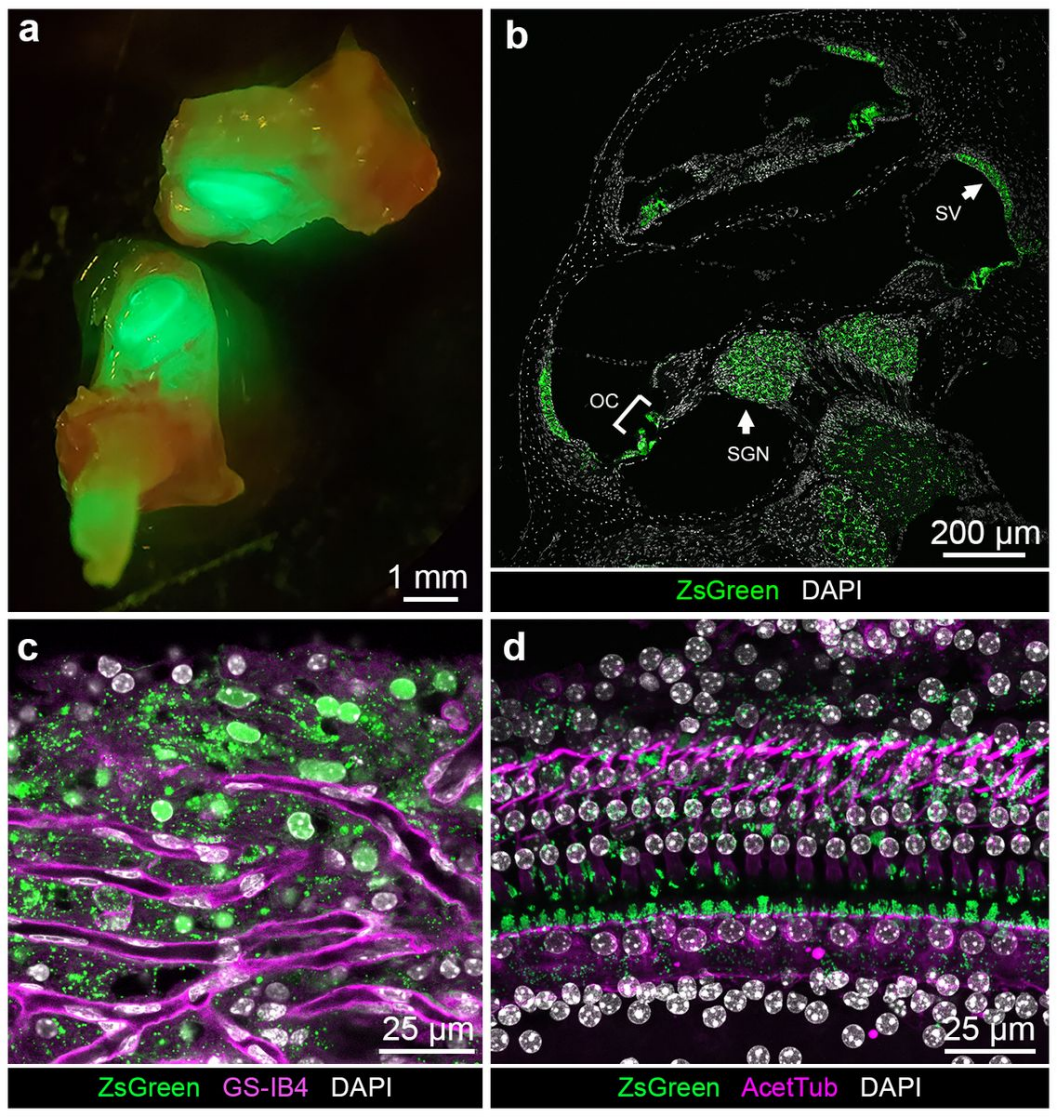
*Kcnj10*-ZsGreen transgene expression in the adult cochlea. **a,** ZsGreen fluorescence was readily visible in the adult inner ear even without microdissection. **b,** Cochlear cross-section in the mid-modiolar region showing ZsGreen fluorescence (green) in the stria vascularis (SV), in the root cells, in a subset of cells of the organ of Corti (OC, bracket), and in the region of the modiolus region containing the spiral ganglion neuron (SGN) cell bodies. The same fluorescence profile was consistently obtained for mice of line 850, similar results were obtained for mice of line 858 (Fig. S1) **c,** Maximum intensity projection of SV whole-mount from mouse analyzed by confocal microscopy demonstrating ZsGreen fluorescence (green) in the region of the intermediate cells, where blood vessel capillaries are recognizable by their isolectin GS-IB4 (magenta) staining. **d,** Maximum intensity projection of confocal image Z-stack of the organ of Corti whole-mount preparation from mouse demonstrating ZsGreen fluorescence (green) between the inner hair cells and outer hair cells in the region of the inner phalangeal cells, the pillar cells and arising around the nuclei of the outer hair cell cells coinciding with the phalangeal processes of the Deiters’ cells identifiable by their acetylated tubulin immunoreactivity (magenta). Additionally, some faint signal was seen in regions corresponding to the inner sulcus/border cells and Hensen’s cells.

To evaluate ZsGreen expression in the cochlea, we examined ZsGreen fluorescence in different settings commonly used for the study of the inner ear in two month old hemizygous mice. *ZsGreen in vivo* fluorescence was readily visualized in the inner ear without amplification even through the bone of the cochlear duct of the mature inner ear as well as in fixed cochlea cryosections (Fig. 3a, b). In low magnification view of a mid-modiolar cross-section of the cochlea, ZsGreen signal was detectable in the SV, in the root cells, in the organ of Corti, as well as in region of the modiolus (Fig. 3b for line 850, Fig. S1 for line 858). ZsGreen fluorescence was present all along the cochlear duct from base to apex. ZsGreen expression in mice from lines 850 and 858 was undistinguishable.

In the organ of Corti whole-mount imaged at the hair cell nuclei level, ZsGreen fluorescence could be seen in supporting cell area, suggestive of expression in the inner phalangeal cells, pillar cells and Deiters’ cells (Fig. 3d). The phalangeal processes of the Deiters’ cells were recognizable by their acetylated tubulin immunoreactivity as previously described^15^. In whole-mount tissue, ZsGreen fluorescence was also detected in a subset of cells of the SV, in a region with strong vascularization as indicated by the presence of endothelial cells labeled by isolectin GS-IB4^16^, consistent with the potential expression of ZsGreen in the intermediate cells (Fig. 3c). Similarly, light sheet microscopy of *Kcnj10*-ZsGreen cochleae demonstrated expression of the fluorescent reporter in the intermediate cells of the SV, in a subset of cells of the organ of Corti, and in cells located in the modiolus (Video S2). Three-dimensional reconstruction from lateral to medial through the lateral wall further confirmed the expression of ZsGreen in SV intermediate cells which lie between the basal and marginal cell layers of the SV (Fig. 1a and Video S2).

### In the stria vascularis, *Kcnj10*-ZsGreen transgene is expressed specifically in intermediate cells.

**Figure 4:**
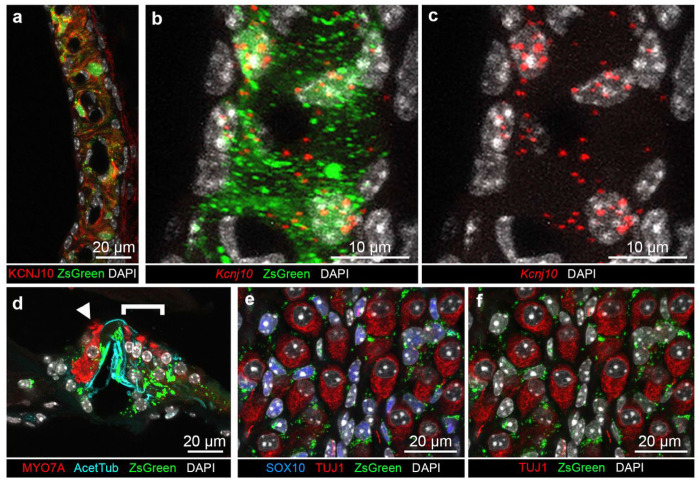
*Kcnj10*-ZsGreen transgene expression in the adult mouse cochlea. **a,** Cross-section of the stria vascularis (SV) showing KCNJ10 protein localization at the plasma membrane (red) surrounding ZsGreen cytoplasmic fluorescence (green). **b,** High magnification view of a cross-section of the SV demonstrating co-expression of ZsGreen (green) and *Kcnj10* mRNA detected by smFISH (red dots) in the same cells, indicative of the expression of ZsGreen in the intermediate cells. **c,** Same image as Fig. 4b showing presence of *Kcnj10*mRNA detected by smFISH (red dots) in intermediate cell nuclei and cytoplasm. **d**In the organ of Corti, ZsGreen fluorescence (green) was present in inner phalangeal cells, pillar and Deiters’ cells, identified by their acetylated tubulin immunolabeling (cyan). Inner hair cells (IHCs, arrowhead) and outer hair cells (OHCs, bracket) identified by their MYO7A immunolabeling, did not show ZsGreen fluorescence. Additionally, some faint signal was seen in regions corresponding to the inner sulcus/border cells and Hensen’s cells. **e,**Cross-section of the modiolus showing ZsGreen fluorescence (green) in cells expressing SOX10, a satellite glial cell nuclear marker (blue). ZsGreen expression was not found in the spiral ganglion neurons immunolabeled with TUJ1 (red). **f,** Same confocal acquisition as Fig. 4e showing that ZsGreen fluorescence did not overlap with TUJ1 labeling. Cell nuclei were labeled with DAPI. Imaging from P60 mice.

As shown in Fig. 4a, ZsGreen was detected in the cells which express endogenously KCNJ10 protein in the SV. ZsGreen fluorescence also overlapped with *Kcnj10* mRNA detected by smFISH (Fig. 4b-c). Thus, the expression of the fluorescent reporter parallels endogenous expression of *Kcnj10* and KCNJ10 in the intermediate cells of the SV allowing them to be readily identified. ZsGreen fluorescence was present in the nuclei and the cytoplasm of these intermediate cells, with some ZsGreen fluorescence present in aggregates in the cytoplasm or subcellular compartments (Fig. 4b). The presence of aggregates may be suggestive of a disturbance in proteostasis as denoted by Blumenstock and colleagues^17^.

### *Kcnj10*-ZsGreen transgene is expressed in pillar cells and Deiters’ cells in the organ of Corti.

In the organ of Corti, ZsGreen fluorescence was not seen in cochlear hair cells identifiable by their MYO7A immunolabeling. ZsGreen fluorescence was detected in a subset of supporting cells. It partially overlapped with the immunolabeling of acetylated tubulin, which is known to be present in both pillar cells and Deiters’ cells where acetylated tubulin is particularly concentrated in their phalangeal processes^15^ (Fig. 4d). ZsGreen fluorescence overlapped with *Kcnj10* mRNA expression detected by smFISH in in the inner phalangeal cells, pillar and Deiters’ cells, with a higher degree of expression in the inner phalangeal cells, and in Deiters’ cells (Fig. S3). The fluorescent reporter appeared to be largely cytoplasmic in these cells. Of note, amongst supporting cells, KCNJ10 protein was also localized in ZsGreen-expressing Deiters’ cells underlying the outer hair cells but did not appear to be present at detectable level in pillar cells unlike *Kcnj10* mRNA (Fig. S4a-d). *Kcnj10* mRNA expression in both pillar and Deiters’ supporting cells was also identified in single cell transcriptome datasets from the P7 organ of Corti^18^ (Fig. S5). The discrepancy between mRNA expression and protein detection may be due to a difference in *Kcnj10* mRNA expression or alternatively, post-transcriptional or post-translational modifications involving KCNJ10 protein preventing its detection by the antibodies we used. Tissue processing involving decalcification and cryopreservation could also influence KCNJ10 epitope accessibility to the antibodies.

### *Kcnj10*-ZsGreen transgene is expressed in satellite glial cells and a subset of spiral ganglion neurons.

In the modiolus, ZsGreen fluorescence was consistently detected in the satellite glial cells as demonstrated by its presence in the cytoplasm and to a lesser extend the nuclei of the cells which express SOX10, a nuclear marker of glial cells^19–22^ (Fig. 4e, Fig. S6a-c). On the contrary, ZsGreen was not detected in most spiral ganglion neurons recognizable by their TUJ1-immunoreactivity (Fig. 4e-f).

*Kcnj10* mRNA expression detected by smFISH was present in both glial cells, as well as at least in a subset of spiral ganglion neuron nuclei (Fig. S6). Corroborating these results, single cell transcriptomic analysis of *Kcnj10* mRNA expression in adult spiral ganglion neuron subtypes (Types IA, IB, IC, II) and satellite glial cells demonstrated its expression in both satellite glial cells and type II spiral ganglion neurons^23^ (Fig. S7). While ZsGreen was detected consistently in glial cells expressing endogenously *Kcnj10* (Fig. S6b-c), its expression was more variable in spiral ganglion neurons, including those expressing endogenously *Kcnj10* (Fig. S6d-f). KCNJ10 immunolabeling was also detected in SOX10-expressing satellite glial cells (Fig. S8a-d) and at least a subset of TUJ1-expressing spiral ganglion neurons (Fig. S8e-h). In these experiments, ZsGreen was also detected consistently in the glial cells, but seemed to be only detectable in a subset of spiral ganglion neurons (Fig. S8).

In summary, ZsGreen transgene expression appears to reflect endogenous *Kcnj10* mRNA and KCNJ10 protein expression in satellite glial cells, with a more variable expression in a subset of spiral ganglion neurons, offering a useful genetic label for the study of these cell types in the future.

### ZsGreen expression does not alter the audiometric profile nor the endocochlear potential of *Kcnj10*-ZsGreen mice.

**Figure 5:**
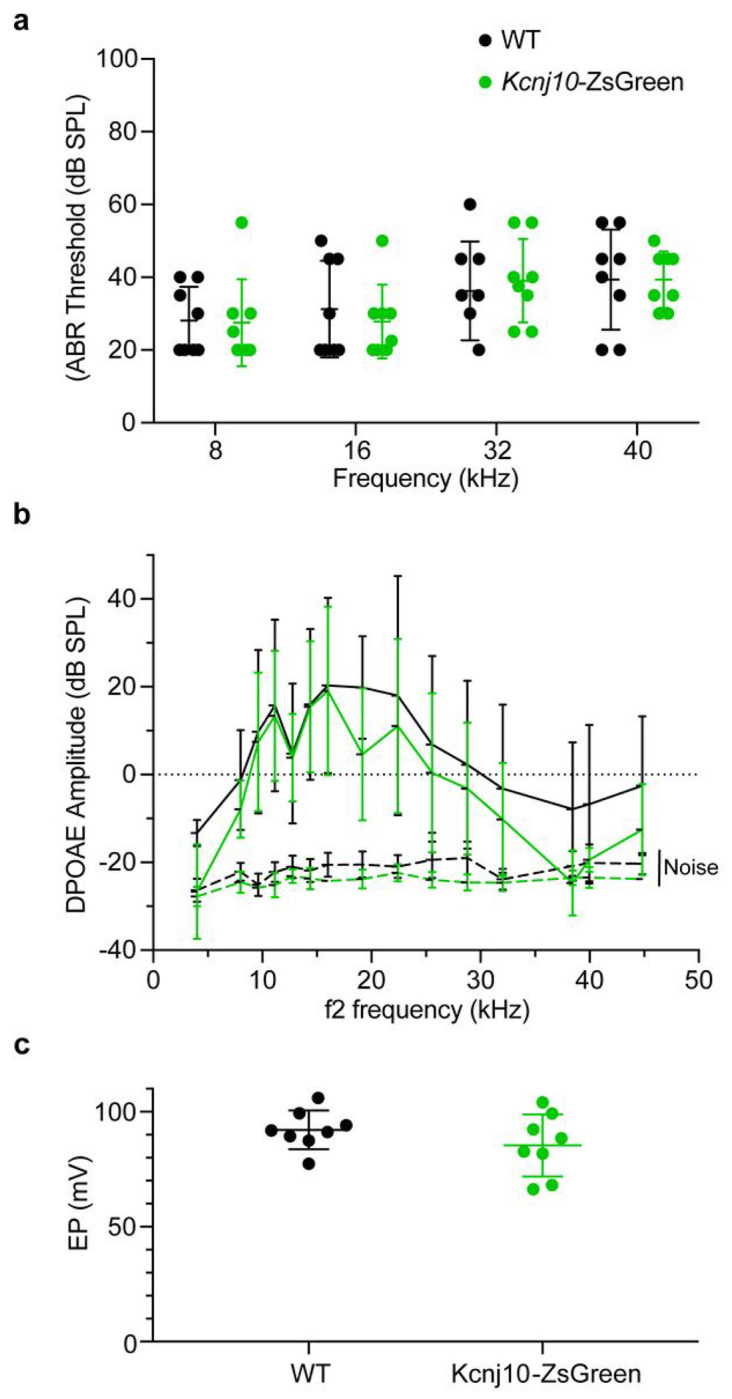
Audiometric profile of *Kcnj10*-ZsGreen reporter mice at P60. **a,** *Kcnj10*-ZsGreen hemizygote mice have similar ABR thresholds as their wildtype littermates. Two-way ANOVA showed no significant differences between genotype and frequency (p=0.90). **b,** The DPOAEs of these mice were also similar at the different frequencies tested. (F2 frequencies 4005, 8011, 9632, 11158, 12779, 14401, 16022, 19169, 22411, 25559, 28801, 32044, 38433, 39959, 44823 Hz). Two-way ANOVA showed no significant differences between genotype and frequency (p = 0.95). **c,** Endocochlear potential (EP) obtained in *Kcnj10*-ZsGreen mice and their wildtype littermates were also not significantly different (p = 0.26). Each dot represents value obtained in one ear. Each mouse was only tested in one ear where ABRs, DPOAEs and EP were measured. Results obtained for *Kcnj10*-ZsGreen hemizygote mice are presented in green, results obtained for their wildtype littermates are presented in black. Results are presented as mean ± SD.

To test whether ZsGreen expression affected hearing and ionic homeostasis in the transgenic mice, auditory brainstem responses (ABRs), distortion product of otoacoustic emissions (DPOAEs) and EP were measured in these mice at P30. ABR of *Kcnj10*-ZsGreen mice (N = 8) and wildtype littermates (N = 8) were measured at 8, 16, 32, and 40 kHz, to assess cochlear function at different positions along the cochlear duct (Fig. 5a). Two-way ANOVA analysis did not detect any significant differences between ABR obtained for the two genotypes at these different frequencies (p = 0.90) (Fig. 5a). As ZsGreen is expressed in the glia cells, which could influence type I auditory neurons function and viability, we quantified the amplitude and latency of the wave I of these ABRs at 80 dB stimulation to test for the presence of potential hidden hearing loss and auditory dyssynchrony (Fig. S9a, b). Two-way ANOVA analysis of these results did not detect significant differences between ABR amplitude and latency for the two genotypes at the different frequencies tested (p = 0.98 and 0.69, respectively). DPOAEs were measured using F2 frequency at 4005, 8011, 9632, 11158, 12779, 14401, 16022, 19169, 22411, 25559, 28801, 32044, 38433, 39959, 28801, 32044, 38433, 39959, 44823 Hz (Fig. 5b). Two-way ANOVA analysis demonstrated no significant differences between DPOAEs for the two genotypes at the frequencies tested (p = 0.95) (Fig. 5b). EP measurements revealed similarly normal EP values between *Kcnj10*-ZsGreen mice (N = 8, 84.39 ± 12.66 mV) and their wildtype littermates (N = 8) (mean = 91.75 ± 7.88 mV) (p = 0.26) (Fig. 5c). These results indicate that these *Kcnj10*-ZsGreen hemizygous mice have normal hearing and EP and are suitable for the study of hearing function and hearing loss as it relates to cell types with endogenous *Kcnj10* expression.

## Discussion

While the role of the stria vascularis in maintaining the EP has been well documented^2,24^ the mechanisms by which specific SV cell types maintain ion homeostasis and EP remains incompletely defined. To further study these mechanisms, tools that allow for the study of specific cell types in the SV are needed. Here we report the generation and cochlear characterization of transgenic mice expressing ZsGreen fluorescent reporter driven by the promoter and regulatory sequences of a gene specifically expressed in intermediate cells of the stria vascularis, *Kcnj10*^2^. We demonstrate that these mice enable the visualization of SV intermediate cells in both live and fixed tissue. We also show that the expression of the reporter does not affect hearing as measured by ABRs and DPOAEs, and does nor affect the EP providing physiological evidence that this reporter mouse is a relevant tool to study the role of the SV in hearing function.

ZsGreen fluorescence in *Kcnj10-ZsGreen* reporter mice recapitulated the endogenous expression of *Kcnj10* as shown by smFISH and single cell data. ZsGreen was expressed in the intermediate cells of the *Kcnj10*-ZsGreen transgenic mouse allowing their readily identification from the other major cell types of the SV in both live and fixed tissue. ZsGreen was also expressed in the pillar cells, Deiters’ cells, satellite glial cells and a subset of spiral ganglion neurons, all of which support normal hearing function. The supporting cells have various functions including formation of the tunnel of Corti by maturation of pillar cells^25^ and the maintenance of hair cell homeostasis by Deiters’ cells^26^. Satellite glial cells provide structural support to neurons and help facilitate neuron to neuron communication^27^, while spiral ganglion neurons transmit auditory information from hair cells to the brainstem^28^.

Other groups have established that using an open vessel-window approach combined with intravital fluorescence microscopy, one can directly visualize the SV in *vivo*^29^. Using the *Kcnj10* ZsGreen reporter mice with this imaging methodology, one could directly observe the intermediate cells of the SV. Presence of intermediate cells detectable through ZsGreen expression could be utilized as a proxy for intermediate cell viability both *in vivo, ex vivo* and in fixed tissue. This has further applications to study intermediate cell physiology in live tissue, including their electrophysiological properties. When combined with auditory physiology measurements such as ABRs and EP intermediate cell viability could be correlated with clinical hearing changes. For example, cell type-specific effects of pharmacologic interventions on intermediate cells, particularly as they relate to intermediate cell morphology and viability beyond cell death could be ascertained utilizing this mouse model for agents including furosemide^30–32^, aminoglycosides^32^, and cisplatin^33–35^. This reporter mouse could also be utilized to assess the impact of known pathogenic genetic variants on intermediate cell physiology. For example, pathogenic variants affecting *GJB2*, are the most common genetic causes of nonsyndromic human sensorineural hearing loss^36^. *GJB2* encodes connexin 26 which is expressed in SV intermediate and basal cells in mice^4,37^ with slightly different distributions in these cell types in humans^38^. Early oxidative stress and metabolic dysregulation have been identified as downstream results of these genetic variants in the SV^36^. If bred with the appropriate mouse model of disease, this reporter mouse offers an opportunity to study the physiological impacts of these pathogenic variants at the cell type-specific level in regions where connexins are expressed including SV intermediate cells as well as pillar and Deiters’ cells^38^.

Limited mouse auditory cell lines are available for research purposes, with the HEI-OC1 cell line being the most used^39^. With respect to the SV in particular, despite SV cell behavior in explant settings having been widely studied^40–43^, SV cell lines remain even more limited. Available options include the SV-k1 cell line, which has been described as having mostly marginal cell like characteristics. While SV-k1 demonstrates the presence of markers specific to strial marginal cells such as Na,K-ATPase α1 and β2 subunits, the line has not been shown to express the marginal cell specific marker, voltage dependent potassium channel KCNE1^44,45^. Considering the SV contains multiple cell populations, there is a need to produce more SV cell lines that reflect the characteristics of the different cell populations to better study the physiology of each cell type. The *Kcnj10*-ZsGreen reporter mice’s unique feature of fluorescently labelled intermediate cells that can be readily seen on whole-mount preparations, opens the door for the creation of a cell line with cell specific markers and *in vitro* testing applications. This includes the study of the effects of novel and repurposed pharmacologic agents for the treatment of hearing loss on the SV identified from other studies including computationally based repurposing studies^4,35,46,47^. While cell lines may not fully replicate the *in vivo* function in the defined microenvironment of the cochlea, the possibility of organoids containing elements of the SV have been suggested by recent work by van der Valk and colleagues^48^ and offer the potential organoid-based *in vitro* testing applications. Finally, the possibility of using this mouse model for targeted fluorescence-activated cell sorting (FACS)-purification of ZsGreen-expressing SV intermediate cells might allow for more in-depth analyses of these cell types, including potential interactions with neighboring immune cells in the intermediate layer of the SV.

## Conclusions

In summary, we report the generation of a BAC transgenic mouse which utilizes the *Kcnj10* promoter and regulatory sequences to drive ZsGreen expression in the intermediate cells of the SV and to a lesser extent in the satellite glial cells of the spiral ganglion region and cochlear supporting cells, a profile which reproduces the endogenous expression of *Kcnj10*. Expression of the fluorescent reporter does not alter auditory physiology as measured by ABRs, DPOAEs and EP. Overall, this *Kcnj10*-ZsGreen transgenic reporter mouse provides a much needed tool to study, both in live and fixed tissue, intermediate cell function.

## Methods

### Ethics declaration and approval for animal experiments.

All animal experiments and procedures were performed according to protocols approved by the Animal Care and Use Committee of the National Institute of Neurological Diseases and Stroke and the National Institute on Deafness and Other Communication Disorders, National Institutes of Health.

### Generation of *Kcnj10-Zsgreen* BAC transgenic mice.

The bacterial artificial chromosome (BAC) clone RP23-157J4 from RPCI – 23 Female (C57BL/6J) Mouse BAC Library (GenBank: AC074311.28) was identified as containing the gene *Kcnj10*. This BAC (obtained from the BACPAC Resource Center located at Children’s Hospital Oakland Research Institute in Oakland, CA) contained 186 kb of mouse gDNA spanning from Chr1:172236604–172422466 (GRCm38/mm10). *Kcnj10* mRNA (NM_001039484.1) spans from Chr1:172341210–172374085. This gene has 2 exons, with a coding sequence starting at the start of exon 2. The transgenic construct based on BAC RP23-157J4 was engineered with minor modifications using methods described in Lee and colleagues^49^ and Zeidler and colleagues^50^. Reporter mice were generated at the Transgenic Animal Model Core of the University of Michigan’s Biomedical Research Core Facilities.

The synthetic donor DNA was produced by PCR amplification of the recombineering plasmid R6K-PGK-ZsGreen. The PCR primers contained 80 nucleotides of homologous genomic sequences that matched the DNA 5’ and 3’ of the desired insertion in *Kcnj10* genomic DNA. The BAC clone and the synthetic donor DNA were combined in DH10B competent bacteria. Introduction of the synthetic donor into the BAC resulted in kanamycin resistance of the bacteria clones containing the recombined BAC. The kanamycin cassette was then removed by induction of FLP recombinase expression, using plasmid pE-FLP (Addgene, #45978). DNA from kanamycin sensitive BACs were analyzed to identify correctly modified BACs by sequencing and enzyme restriction. A final recombination step replaced the BAC-backbone internal loxP site with ampicillin resistance cassette.

The modified BAC was sequenced to verify that the intended insertion of ZsGreen occurred, using 1350 bp amplicon obtained with primers located 5’ and 3’ of ZsGreen: 5’-CCACCACCTCCAACATGAAT-3’ and 5’-CTCTCTTTCCCCCAAGCTG-3’ and GoTaqGreen polymerase (Promega) with an annealing temperature of 55°C. The final BAC showed a silent (A > G) mutation in the amino acid Lysine at position 15 of ZsGreen. The absence of gross recombination inside the BAC was checked by restriction enzyme digestion followed by pulsed field gel.

This recombinant RP23-157J4 BAC was microinjected into fertilized eggs obtained by the mating of B6SJLF1/J female mice with B6SJLF1/J male mice (stock number #100012) obtained from the Jackson Laboratory (Bar Harbor, ME, USA) at the Transgenic Core Facility of the University of Michigan.

Four transgenic founder mice were obtained by random integration of the transgene in their genome. Genomic DNA prepared from tail or ear clips using the Maxwell 16 System (Promega, Madison, WI, USA) was used to identify the mice carrying ZsGreen (Table S1). Four independent transgenic lines were obtained. After maintaining these for 5 generations and noticing no differences in ZsGreen expression pattern between lines, two of them were cryopreserved and the two remaining, lines 850 and 858, were maintained alive and further backcrossed. Male mice hemizygous carrier of the ZsGreen transgene were mated with C57BL/6J wildtype female mice (stock number #000664) obtained from the Jackson Laboratory for over 10 generations leading to obtention of congenic mice carrier of the transgene. The mice studied here were from the 11th generation and were all hemizygous for the transgene. The resulting mouse strains was named *B6.Cg-Tg(Kcnj10-ZsGreen)^skMHa^* and *B6.Cg-Tg(Kcnj10-ZsGreen)^skMHb^* for lines 850 and 858, respectively. Location of the primers utilized to detect the *Kcnj10*-ZsGreen transgene in mice are shown in Fig. 2a. Primer sequences and PCR conditions used for genotyping are available in Table S10.

### Single-cell and single-nucleus RNA-sequencing dataset analysis

Adult spiral ganglion neuron and satellite glia cells and P7 organ of Corti single cell RNA-Seq datasets and adult SV single-nucleus RNA-Seq datasets from the mouse were analyzed for expression of *Kcnj10*^3,18,23^. Violin plots of expression among cell types in the SV (marginal, intermediate, basal and spindle cells), cell types in the P7 organ of Corti (inner and outer hair cells, pillar cells, and Deiters’ cells) and in the spiral ganglion region (Type IA, IB, IC and type II spiral ganglion neurons, satellite glial cells) were constructed as we have previously described^3,4^.

### Reporter transgene copy number assessment.

To assess the number of transgene copies integrated in the gDNA of the transgenic mice, digital droplet PCR (ddPCR, Bio-Rad, Hercules, CA, USA) was performed. Seven animals (4 males and 3 females) from each of the two founder lines (850 and 858) were tested in *Kcnj10*-ZsGreen group. Five wildtype littermates were included as control. gDNA was isolated from tail snip using Maxwell 16 DNA purification system (Promega, RRID:SCR_020254), DNA concentration was measured using Thermo Scientific NanoDrop One/OneC Microvolume UV Vis Spectrophotometer (Thermo Fisher Scientific, Waltham, MA, USA, RRID:SCR_023005) and was adjusted to 50 ng/uL. The autosomal gene *Actb* was used as reference. In ddPCR reaction, each well contained 1 uL of ZsGreen probe (FAM labeled)/primers mix, 1 uL of the probe recognizing the gene *Actb* (HEX labeled)/primers mix, 1uL of gDNA (50 ng), 10 uL Bio-Rad ddPCR Supermix (Bio-Rad, Hercules, CA, USA, Cat#1863024) for Probes and 7 uL molecular grade water. The ddPCR Droplets were generated using the QX200 AutoDG Droplet Digital (Bio-Rad, Cat#1864101). PCR was performed as described in the QX200 ddPCR EvaGreen Supermix instructions. Droplets were read with a QX200 Droplet Reader (Bio-Rad, Cat#1864003) and analyzed with QuantaSoft software (Bio-Rad, Cat#1864011). Sequences of the primers and probes used for this experiment are presented in Table S11.

### Tissue preparation for immunofluorescence labeling and *in situ* hybridization.

Hemizygous transgenic mice and their wildtype littermates were studied. Unless indicated otherwise mice were studied at P56 to P65 (summarized here at P60) for all experiments. Inner ears from these mice were dissected and placed in 4% paraformaldehyde (PFA in 1 x PBS solution) overnight at 4°C. Fixed adult mouse inner ears were then decalcified in 0.25 M EDTA for 3 days in 4°C on orbital shaker. If the tissue was designated for immunostaining or hybridization as whole mounts, it was washed in PBS, dissected and stored in PBS in 4°C for further use. If the tissue was designated for cryosections, it was washed in PBS, transferred to 30% sucrose in PBS at 4°C overnight, followed by immersion in 50/50 mix of 30% sucrose in PBS and finally in super cryo-embedding medium (SCEM) (C-EM001, Section-Lab Co, Ltd.; Hiroshima, Japan). Tissue was flash-frozen in liquid nitrogen after the transfer and 2-hour incubation in fresh 100% SCEM in a cryomold biopsy square.

Fluorescent immunohistochemistry was performed as follows. Cryosections or whole mount tissue were washed in PBS then permeabilized and blocked (2 hours at room temperature (RT) for cryosections, overnight at 4°C for whole-mounts) in PBS with 0.2% Triton X-100 (PBS-T) with 10% fetal bovine serum (A3840001, ThermoFisher Scientific, Waltham, MA, USA). Samples were then incubated in the appropriate primary antibodies in PBS-T with 10% fetal bovine serum for 24 hours in 4°C, followed by two 10-minute washes in PBS-T and labelling for 2 hours at RT with AlexaFluor 488, 555 and/or 647-conjugated secondary antibodies made in donkey and directed against appropriate species (Life Technologies, Waltham, MA) diluted at 1:250 in PBS-T. DAPI (4,6-diamidino-2-phenylindole,1:10,000, Life Technologies, Waltham, MA) was included with the secondary antibodies to detect nuclei. Alexa Fluor 647 phalloidin was used to label F-actin in a subset of experiments. Samples were washed in PBS four times for five minutes and mounted in SlowFade Gold (S36937, Invitrogen, ThermoFisher). Specimens were imaged using Zeiss LSM880 confocal microscopes (Zeiss, Oberkochen, Germany) using 40x, 1.4 numerical aperture and 63x, 1.4 numerical aperture objectives. Primary antibodies utilized are detailed in Table S12.

### Single molecule fluorescent *in situ* hybridization (smFISH).

*In situ* hybridization on PFA fixed, frozen tissue was performed with RNAscope^®^ Multiplex Fluorescent Reagent Kit v2 (Advanced Cell Diagnostics, Hayward, CA, USA) with substantial modification of pretreatment process was performed as previously described^3,4,35^. Briefly, frozen sections on Superfrost Plus microscope slides were removed from − 80°C freezer, placed on heating block (37°C) for 30 minutes to thaw and dry, then moved to RT. Hydrogen peroxide solution was applied on the section and incubated for 10 minutes at RT. Slides were washed 2 times in deionized water, dried initially on heating block for 10 minutes at 37°C, then placed on heating block and baked for 30 minutes at 60°C. After the baking, slides were moved to RT and hydrophobic barrier was applied around the specimen and left to dry. After 10 minutes, Protease PLUS was applied on the sample and slide was moved to hybridization oven set to 40°C and incubated for 25 minutes. Then slides were washed twice in deionized water, appropriate probe was applied to cover the sample and slides were incubated in hybridization oven for 2 hours at 40°C. Next steps of the protocol directly followed RNAscope^®^ Multiplex Fluorescent Reagent Kit v2 User Manual (Doc. No. 323100-USM).

### Light sheet microscopy imaging of *Kcnj10*-ZsGreen adult mouse cochlea.

#### Sample processing

Mouse cochlea fixed in 4% paraformaldehyde was washed in PBS at RT for half a day and then transferred into 50 mL of decalcification solution (20% EDTA in 1X PBS, pH = 9) for 7–10 days at 40°C. Fresh decalcification solution was supplied every 3 days. Finally, the decalcified cochlea was washed in PBS for 1 day at 4°C. To clear the tissue, decalcified mouse cochlea was dehydrated in a step gradient of tetrahydrofuran/water solutions (20%, 40%, 60%, 80%, 100% tetrahydrofuran) at 4°C. The duration of each step was 12 h and the cochlea was incubated in 100% tetrahydrofuran one extra time at 4°C overnight. Dehydrated cochlea was incubated in 100% dibenzyl ether at 4°C until the sample sank and was incubated one more time in fresh 100% dibenzyl ether at 4°C overnight before imaging.

#### Cleared tissue DISPIM

The dual-view inverted selective plane illumination microscope optimized for cleared tissue (CT-DISPIM; Applied Scientific Instrumentation, Eugene, OR, USA), and the associated data processing pipeline have been described in detail previously^51^.

We used a pair of 0.7 N.A. multi-immersion objectives (Special Optics; Denville, NJ, USA) to acquire images of a cochlea that had been cleared using iDISCO and mounted in dibenzyl ether (DBE). The sample was excited by a digitally scanned OBIS laser (Coherent; Santa Clara, CA, USA) light sheet. Fluorescence was filtered through an emission bandpass filter before being recorded on a Hamamatsu Flash 4 v3 sCMOS camera (Hamamatsu Photonics; Shizuoka, Japan). *Kcnj10*-ZsGreen was imaged using 488 nm excitation and a 525/50 bandpass emission filter. Autofluorescence was imaged using 637 nm excitation and a 676/37 bandpass emission filter (both filters from Semrock; West Henrietta, NY, USA). Image volumes were acquired in single view mode as stage-scanned tiles at full frame (2048 × 2048 pixels, FOV 520 μm, 0.254 μm per pixel) with 1 μm perpendicular inter-plane distance and 10% overlap between adjacent tiles. Image tiles were then stitched and deskewed on the NIH Biowulf supercomputer. Three-dimensional rendering was done in Imaris (version 9.9, Oxford Instruments).

### Auditory brainstem responses and distortion product otoacoustic emissions.

Auditory brainstem responses (ABRs) were detected in both ears of anesthetized mouse at age P60-P65. Wildtype littermates lacking the transgene were used as a control group. Mice were anesthetized with an intraperitoneal injection of ketamine (56 mg/kg) and dexdomitor (0.375 mg/kg) and placed on a heating pad connected to a temperature controller (TC-2000, World Precision Instruments, Sarasota, FL, USA) inside a sound-attenuated booth (Acoustic Systems, ETS-Lindgren, Austin, TX, USA) to maintain animal body temperature at 37°C. Recordings were obtained using Tucker-Davis Technologies (Alachua, FL, USA) hardware (RZ6 Processor) and software (BioSigRZ, version 5.7.5). For ABR testing, subdermal electrodes (Rhythmlink, Columbia, SC, USA) were placed at the vertex, under the test ear, and under the contralateral ear (ground). Blackman-gated tone burst stimuli (3 ms, 29.9/s, alternating polarity) were presented to the test ear at 8, 16, 32, and 40 kHz via a closed-field Tucker-Davis Technologies MF-1 speaker. Responses were amplified (20×), filtered (0.3-3 kHz), and digitized (25 kHz) with 512–1024 artifact-free responses per waveform. For each frequency, testing began at 80 dB SPL and decreased in 10 dB steps until the ABR waveform was no longer discernable. Once the response was lost, testing continued in 5 dB steps with a minimum of two waveforms per stimulus level to verify repeatability of ABR waves. ABR thresholds were determined by visual inspection of stacked waveforms for the lowest stimulus level that yielded repeatable waves.

Distortion-product otoacoustic emissions (DPOAEs) were measured in both ears using Tucker-Davis Technologies hardware (RZ6 Multi I/O processor, MF-1 speakers) and software (BioSigRz, version 5.7.5) in conjunction with an Etymotic ER-10B + microphone. Two tones were presented simultaneously at levels of f1 = 65 dB SPL and f2 = 55 dB SPL with the higher frequency tone (f2) set between 4-44.8 kHz (5 points per octave) and f2/f1 = 1.25. Mean noise floors were calculated from levels at six frequencies surrounding the 2f1-f2 DPOAE frequency.

### Endocochlear potential measurement.

Methods for EP measurement have been described previously^52–55^. Briefly, P63-P68 mice were anesthetized with 2,2,2-tribromoethanol (T4842, Sigma-Aldrich, St. Louis, MO, USA) at a dose of 0.35 mg/g body weight. EP measurements were made using glass microelectrodes inserted into the round window and through the basilar membrane of the first turn of the cochlea. Induction of anoxia, allowing measurement of anoxic-state EP was accomplished by intramuscular injection of succinylcholine chloride (0.1 μg/g, NDC-0409-6629-02, Pfizer, New York, NY, USA) after establishment of deep anesthesia followed by additional injection of 2,2,2-tribromoethanol. Anoxic-state EP provides an indicator of the lowest EP and sensory hair cell function. In the presence of functional hair cells, the anoxic-state EP is negative, whereas the EP is zero if the hair cells are not functional. Data were recorded digitally (Digidata 1440A and AxoScope 10; Axon Instruments) and analyzed using Clampfit10 (RRID: SCR_011323, Molecular Devices, San Jose, CA, USA). Eight *Kcnj10*-ZsGreen transgenic mice and eight wildtype C57BL/6J littermates were evaluated.

## Statistical Analysis

Results of quantifications are presented as mean ± standard deviation (SD). For pairwise comparisons of ABR and DPOAE data between *Kcnj10*-ZsGreen mice and wildtype littermates, two-way ANOVA was conducted. For pairwise comparison of EP between *Kcnj10*-ZsGreen mice and wildtype littermates, an unpaired t-test with Welch’s correlation was used. All statistical analysis were performed using GraphPad Prism version 8.4.3 (GraphPad Software, San Diego, CA, USA).

## Data Availability

The authors confirm that the data supporting the findings of this study are available from the corresponding author MH on request. Transgenic mice will be made available upon reasonable request and are in the process of being deposited at The Jackson Laboratory.
